# Supplementary material for: Are carbon emissions trading and green financial instruments synergistic? -Comprehensive quantitative research based on content analysis
Source: PLoS One. 2024 Mar 7;19(3):e0298601. doi: 10.1371/journal.pone.0298601 (PMC10919601; doi:10.1371/journal.pone.0298601)
Supplement: S1 Data — (ZIP) [file pone.0298601.s001.zip › Data/地级市Z_F工作报告.zip!/╔╜╢1⁄2/┴─│╟╩╨/└┤╫╘╣1⁄2╓┌║┼í╛┬φ┐╦╩2╛▌═°í┐_2012.docx]

各位代表：

　　现在，我代表市人民政府向大会报告工作，请予审议，并请市政协委员提出意见。

　　一、过去四年工作的回顾

　　市十五届人大一次会议以来的四年，是我市在复杂形势下抢抓发展机遇、在严峻挑战面前继续开拓前进的四年，是全市上下团结奋斗、经济社会事业全面发展实现新突破的四年。四年来，在中共聊城市委的正确领导下，我们坚持以邓小平理论和“三个代表”重要思想为指导，深入贯彻落实科学发展观，认真执行中央、省委省政府的各项决策部署，紧紧围绕加快建设生态型强市名城实现新跨越的奋斗目标，抢抓机遇、攻坚克难、奋力开拓，全面完成了“十一五”规划目标任务，实现了“十二五”良好开局，全市经济建设、政治建设、文化建设、社会建设和生态文明建设都取得新的重大成就。

　　（一）过去的四年，是我市战胜前所未有的困难和挑战，综合经济实力迈上新台阶的四年。四年来，国际国内形势跌宕起伏、复杂多变，特别是国际金融危机对我市经济社会发展造成严重冲击。面对复杂形势和重大风险挑战，我们坚持化危为机、弯道超越，紧紧扭住科学发展、跨越发展不放松，组织开展了“落实年”、“项目突破年”、“全面提升年”、“继续提升年”活动，全市经济保持平稳较快发展，主要经济指标增幅均高于全省平均水平。2011年，全市生产总值达到1905.2亿元，是2007年的1.6倍，年均增长12.9%，高于全省平均水平1.1个百分点，人均生产总值迈上3万元台阶；地方财政收入96.6亿元，是2007年的2.3倍，年均增长22.9%,高于全省平均水平3个百分点；社会消费品零售总额630亿元，是2007年的1.95倍；本外币存贷款余额分别达到1366.6亿元、1110.7亿元，是2007年的2倍和1.9倍。各县（市、区）竞相发展，茌平跨入全国百强县行列。

　　（二）过去的四年，是我市抢抓机遇、加大投入，基础设施和发展后劲取得新突破的四年。我们始终坚持把抓投入、抓项目作为保增长、转方式、调结构的关键，千方百计加大优质高效投入，坚定不移地上大项目、上好项目。金融危机期间，坚决贯彻中央宏观调控政策，抢抓国家实施刺激经济增长一揽子计划等机遇，开展“项目突破年”活动，实施“产业提升工程”，项目、土地和资金争取工作一举扭转了以往被动局面，四年来均达到全省平均数以上。坚持每年抓好100个重点项目，累计完成投资1395.5亿元，信发集团化工配套及赤泥处理、祥光铜业40万吨阴极铜、鲁西化工精细化工、中色奥博特20万吨铜合金板带、时风集团20万辆电动汽车、中冶银河20万吨高档文化用纸、泉林纸业80万吨有机肥等一批大项目陆续建成、发挥效益。交通、能源等基础设施建设取得重大突破，中华电力2×60万千瓦机组建成投产，京九铁路聊城段电气化改造、聊城火车站改造全面完成，高临高速公路主线完工，邯济铁路扩能改造、德商高速、济聊一级公路、聊城国家公路运输枢纽正在建设，四年新建改建干线公路318公里，市区与各县（市）之间实现由一级以上公路连接。可以说，聊城发展的基础条件正在发生根本性变化。

　　（三）过去的四年，是我市着力转方式、调结构，各产业发展开创新局面的四年。我们坚持把保增长与转方式、调结构相结合，以打造“一五二”产业基地为重点，努力培植产业发展新优势。一是推进农业产业调整振兴。坚持三农稳市，在全省率先提出实施了《农业产业调整振兴计划》。粮食生产实现“九连增”，总产连续登上70亿斤、80亿斤、90亿斤、100亿斤四个台阶，2011年达到107.5亿斤，市及8县（市、区）全部受到国务院或省政府表彰，是全省唯一的市，我们用不到全国1‰的土地，生产了全国1%、全省1/8的粮食，为国家粮食安全作出了突出贡献。蔬菜和食用菌、畜牧和水产、林果生产及加工产值分别达到150亿元、140亿元和160亿元，全市农业综合生产能力迈上一个大的台阶。省级以上农业龙头企业达到34家，农民专业合作社4151个，分别是2007年的2倍和45倍；三品认证品种266个，基地面积315.6万亩，分别是2007年的2.6倍和1.3倍。四大灌区续建配套和节水改造、南水北调聊城段、全国小农水重点县建设、中小河流治理扎实推进，新打、修复机井2.8万眼，旱涝保收面积占到总耕地面积的78%。农业机械化水平达到78.5%，初步把农民从繁重的体力劳动中解放出来。二是推进工业做大做优。坚持工业强市，制定并实施了十大产业调整振兴规划，大力推进 “4455”工程建设。四大“千亿产业园区”发展框架全面拉开，我市被国家有关部门命名为“中国有色金属新城”，被省政府认定为“新能源汽车示范基地”；新口径高新技术产业产值占规模以上工业比重达到14.9%；民营经济占生产总值比重达到51.5%；主营业务收入过百亿元的企业达到6家。四年来，我市连续三年获得省长质量奖，为全省连续获奖的两个市之一；规模以上工业主营业务收入突破5000亿元大关，由2007年的全省第12位一举跃居第7位。这标志着我市新型工业化进程实现了历史性突破！三是推进服务业跨越发展。坚持三产兴市，加快商贸物流、文化旅游、金融保险和房地产业发展，狠抓重点项目建设，推进企业非核心业务剥离。2011年，全市4A级景区达到4个，旅游总收入达到64.3亿元，四年年均增长23%；服务业占生产总值比重达到30.2%，比2007年提高4.3个百分点。四是推进科技创新和节能减排。新增国家重点实验室、工程技术研究中心和企业技术中心7家，获省科技进步一等奖6项，被认定为有色金属材料及制品国家火炬计划特色产业基地、国家知识产权试点城市，成功创建国家级高新技术创业服务中心。同高校院所合作进一步加强，西安交大聊城科技园正在建设，快速制造中心在聊城设立了示范中心。大力实施人才强市战略，建成博士后流动工作站8个。生态文明市建设迈出重大步伐，节能和减排在全省的位次由2007年的16位分别提高到第6位和第4位，代表山东省首次参加全国海河流域水污染防治考核并取得第一名，在环保部采用新标准后第一个成功创建国家环保模范城市。各位代表，四年来我市农业经济走在全省前列，工业经济达到全省中上游水平，服务业占生产总值比重首次突破30%，科技进步进入全国先进行列，在节能和减排上双双打了漂亮的翻身仗。这充分表明，我市转方式、调结构迈出重大步伐，经济素质和产业竞争力实现了新跨越！

　　（四）过去的四年，是我市拉开城市框架、加强新农村建设，城乡面貌展现新气象的四年。我们坚持以加快新型城镇化进程为目标，进一步加大了城乡建设力度，城镇化水平提高了6.5个百分点，比全省同期快2.5个百分点。高水平制定了《全市土地利用总体规划》、《城市空间战略规划研究》、《城市建筑高度分区规划》等专项规划和一批重点项目控制性详规。坚持“城建靓市”，四年累计完成城市建设开发投资739亿元，其中2011年达到214亿元，是2007年的5倍。按照“江北水城·运河古都”的城市定位，加强中心城区建设，新建改建城区道路28条，建成区面积由59.9平方公里增加到68.1平方公里，城区人口由51万人增加到62万人；新增集中供热面积315万平方米、管道燃气居民用户5.6万户，有线数字高清电视在全国地级市中率先实现城区全覆盖；中华水上古城独特风貌已具雏形，徒骇河世界运河（建筑）博览园建设加快推进，东昌湖风景区、运河四期、体育公园全运场馆等工程全面完成，一批设计漂亮新颖的标志性建筑、高层建筑先后建成，水城特色进一步凸显。2008、2009年度分别被评为中国“十大特色休闲城市”、“十大宜居城市”。各县（市）城区和小城镇建设也都取得很大进展，城镇面貌发生了显著变化。坚持统筹城乡发展，新改建农村公路2886公里、客运站112个，改造农村危桥277座，所有行政村实现通油路、通客车，县到乡的客运线路公交化改造基本完成；新建沼气池9.8万个，新增64万人用上自来水，实现了村村通有线电视；农村新居建设和危房改造“三年任务、两年完成”，启动千户社区86个、整村改造300个，建成新房22.1万户，改造危房3.7万户，60多万农民乔迁新居，农村基础条件、农民生活环境进一步改善。各位代表，在成功创建国家优秀旅游城、双拥模范城之后，我们又成功完成了国家卫生城、国家环保模范城、国家园林城的创建工作，市委、市政府超前决策、全市人民共同期待的“五城同创”任务全面完成！

（五）过去的四年，是我市深入推进改革、全面扩大开放，经济社会发展不断增强新活力的四年。我们坚持把改革开放作为实现科学发展、跨越发展的强大动力，始终高度重视、积极推进。国有资产监管体制框架基本建立，市属经营性国有资产监管实现全覆盖。鲁西化工与鼎辉投资成功合作，昌润集团与鑫亚公司实现优势互补、强强联合。鲁西化工实施了定向增发,华泰化工、纷美包装分别在创业板和香港上市。农村承包土地流转57.5万亩，集体林权制度改革圆满成功。市县两级政府机构改革全面完成，分类推进事业单位改革工作已经展开。积极走出去、请进来，举办了2010海内外企业家齐鲁行等一系列招商活动，提出实施了“海外聊城”战略，与华润、中色、中冶、中盐等知名央企成功对接。四年累计招商引资到位资金1026.2亿元，利用外资4.26亿美元；2011年进出口总值达到55.9亿美元，是2007年的3.3倍，年均增长34.5%；累计完成境外投资2.65亿美元，取得探矿权1.04万平方公里，储备铝土矿6.7亿吨。去年，我们组织开展了春、秋两季招商引资“百日会战”，落地项目778个，到位资金405亿元，均创历年之最，聊城对外开放工作开始呈现出全新局面。

　　（六）过去的四年，是我市社会事业全面进步、人民群众生活水平得到新提高的四年。我们坚持以人为本，在财政比较困难的情况下，不断加大民生投入，2011年全市财政用于民生的支出达到113亿元，占财政支出的65%，高出全省10个百分点。全市城镇居民人均可支配收入达到20649元，农民人均纯收入7735元，均为2007年的1.7倍。四年新增城镇就业35.8万人，城镇登记失业率控制在3.3%以内。全面建立了农村低保制度、农村五保财政供养制度；城乡居民养老、医疗保险实现全覆盖，全市70多万60岁以上居民全部领到了基础养老金；“老工伤”人员全部纳入工伤保险，关闭破产企业离退休人员医疗保险保障问题基本解决，统筹城乡、覆盖全民的社会保障体系初步形成。4次提高城乡低保标准和企业退休人员养老金，新农合和城镇居民医疗保险政府补助标准提高到每人每年200元，保障水平进一步提高。城市住房保障制度全面实行，实施廉租住房补贴4954户，开工建设廉租住房3958套，建设经济适用房9576套，棚户区改造14933户，启动建设公共租赁房3609套、限价房2750套。各级各类教育均衡协调发展，教学质量明显提高。全市中小学全部免除了杂费，农村中小学免除了课本费，实现了真正意义上的义务教育。建立了从小学到大学的贫困生资助体系，困难家庭子女上学问题得到较好解决。新建重建校舍67.4万平方米，农村中小学面貌发生了显著变化。实施了城区小学改扩建计划，新建教室122个，小学班额全部控制在56人以内。2011年大学本科录取人数列山东省第3位。市直中等职业教育资源整合取得重大突破，聊城高级财经职业学校、高级工程职业学校新校区正在建设。各类高校再上新水平。卫生事业改革发展取得明显成效，城市社区卫生服务中心、卫生服务站和乡镇中心卫生院、村卫生室建设改造任务全面完成；启动实施了基本药物制度，东阿县公立医院改革走在全省前列；创造性开展了农民健康服务行动，受惠农民34万人，为农民节约医疗费用1140多万元。文化事业发展繁荣，市民文化中心、市豫剧院正在建设，中国运河文化博物馆完成布展并免费开放，县（市）新建文化场馆11座，综合文化站、农家书屋分别实现了乡镇、村级全覆盖。体育工作取得新成绩，新建全民健身站点1500多处，成功承办了十一届全运会部分赛事。人口和计划生育工作进入全省先进行列，年均人口自然增长率为5.98‰。精神文明和民主法制建设进一步加强，荣获全国双拥模范城“三连冠”、全国民族团结进步模范集体，村居两委换届选举圆满完成。食品药品安全动态监管机制初步建立，安全生产事故各项指标全面下降，平安奥运、平安全运、平安世博等重大安保任务圆满完成，社会保持和谐稳定。支援四川地震灾区任务圆满完成。新闻出版、社会科学、妇女儿童、青少年、慈善、残疾人、老龄事业持续发展，外事侨务、宗教、档案、史志、气象、油区、人防、双拥、防震减灾、对台等工作都取得了新的成绩。

　　（七）过去的四年，是我市加强政府自身建设与创新，向全市人民展现新面貌的四年。我们坚持依法行政、从严治政，努力转变政府职能，推进管理创新。四年来，认真执行人大及其常委会的决议、决定，自觉接受人大、政协、群众和舆论监督，办理人大代表建议和政协委员提案953件。精减行政许可71项，取消行政事业性收费40项；市行政服务中心作为全国唯一标准化试点顺利通过验收，荣膺“国家级服务业标准化示范单位”。认真办好市长公开电话、行风热线和社情民意调查，加大信访、仲裁工作力度，一大批涉及群众实际利益的问题得到较好解决。推进政务信息化，“数字聊城”建设走在全国地级市前列，市政务网站跻身全国地级市50强。全面推进政务公开，严肃查处十三种不作为、乱作为行为，发展环境明显优化。加强政府应急工作，应对突发事件的能力不断增强。强化行政监察和审计监管，严肃查处了一批违法违纪案件。深入实施政府部门目标责任管理，出台了转变作风、狠抓落实“八项制度”，充分调动起广大政府工作人员的积极性和主观能动性，全市上下精神面貌焕然一新，狠抓落实蔚然成风。

　　各位代表，过去四年我们面临的复杂形势、遇到的困难挑战前所未有。2008年前三季度，我市经济运行面临煤电油运和资金紧张；之后，国际金融危机对我市经济社会发展造成严重冲击；2010年以来，土地、节能、环保等又成为制约因素。同时，四川汶川特大地震灾害需要我们大力支援，北京奥运会、上海世博会、第十一届全运会需要我们全力保障，手足口病、甲型H1N1流感疫情需要我们科学应对。在巨大困难和严峻挑战面前，我们不仅没有放慢发展的步伐，而且实现了经济平稳较快发展、产业结构进一步优化、发展后劲显著增强、社会民生明显改善，这是非常不容易的。这是中央和省委省政府审时度势、科学决策的结果，是中共聊城市委创新思路、坚强领导的结果，是人大、政协和社会各界有效监督、大力支持的结果，更是全市人民同心同德、艰苦创业、扎实苦干的结果。在此庄严时刻，我代表市人民政府，向全市工人、农民、知识分子，向所有企业家和纳税人，向各民主党派、工商联、各人民团体和各界人士，向驻聊人民解放军、武警官兵、公安干警和中央、省驻聊单位，向所有关心支持聊城发展的海内外朋友，表示衷心的感谢！

　　各位代表，近年来，聊城经济社会发展取得了巨大成就，但仍然存在一些矛盾和问题，主要是：经济总量依然偏小，人均水平较低，财政实力较弱；产业结构还不够合理，发展方式较为粗放；经济外向度低，科技创新能力不强；城镇化进程滞后；群众生产生活还存在一些亟待解决的问题；各级的工作执行力和服务发展的水平需要进一步提升，观念、体制、人才的制约有待进一步破解等等。对此，我们一定要高度重视，采取有效措施，认真加以解决。

　　二、今后五年的目标任务

　　各位代表，最近，中共聊城市第十二次代表大会胜利闭幕，会议确定了今后五年“全面建设生态型强市名城，为创造聊城人民幸福生活而奋斗”的中心任务。这一中心任务的确定，充分体现了聊城人民的共同期盼，完全符合聊城实际，是市委站在新的历史起点上作出的重大战略决策，也是我们必须承担起来的庄严使命和历史责任！

各位代表，综观今后五年面临的形势，机遇与挑战并存，机遇大于挑战。从国际上看，国际金融危机的影响远未结束，国际经济形势复杂严峻，不确定、不稳定因素增多，但和平发展合作仍是时代潮流。从全国看，我国经济长期向好的基本面没有变，仍处在大有作为的重要战略机遇期。中央在继续优化东部地区发展的同时，更加注重中西部地区发展，聊城作为山东东引西拓的“桥头堡”，将面临新的发展机遇。从全省看，我省大力实施“蓝”、“黄”两大战略，加快省会城市群经济圈发展，对我市予以重点支持，启动以济南为中心的城际铁路规划建设，将给我们创造和提供新的有利条件。从我市看，我们正处于工业化、城镇化快速推进的关键时期，一批在全国同行业领先的重点企业、现代产业蔚然兴起，经济总量迈上千亿级台阶，经济效益达到百亿级规模，基础设施日臻完善，城市影响力日益增强，体制机制更具活力。周边各市竞相发展、竞争激烈，对我们既是挑战，更是动力。特别是经过改革开放多年的磨练和考验，我市成长起一大批高素质的领导干部、优秀企业家和专业技术人才，全市上下形成了政通人和、人心思上的浓厚氛围，我们完全有条件加快发展步伐，推动经济社会发展再上新台阶。总之，我们既面临着不少困难和挑战，更面临着难得的机遇和条件，我们对实现争先进位、跨越发展充满了信心和决心！

　　今后五年，政府工作的总体要求是：高举中国特色社会主义伟大旗帜，以邓小平理论和“三个代表”重要思想为指导，深入贯彻落实科学发展观，以科学发展为主题，以加快转变经济发展方式为主线，认真贯彻市第十二次党代会精神，积极作为、科学务实、稳中求进、争先进位，继续坚持“四市方针”，着力打造“一五二”产业基地，在结构调整、节能减排、改革创新、惠民富民、文化繁荣、和谐稳定等方面实现新突破，努力把聊城建设成为山东西部的新兴生态化工业城市、冀鲁豫交界地区的商贸物流中心城市、江北文化旅游和休闲度假目的地城市，为全面建设生态型强市名城实现新跨越，为创造聊城人民的幸福生活打下更加坚实的基础。

　　——今后五年，我们要着力推动科学发展跨越发展，实现经济实力大提升。坚持把发展作为第一要务，在发展中求转变、在转变中谋发展，切实做到好字当头、好中求快、又好又快，确保主要经济指标增幅高于全省平均水平、高于周边市。全市生产总值年均增长13%，地方财政收入和固定资产投资年均增长18%。建成具有较大影响力和竞争力的“一五二”产业基地，即：在一产方面，打造生态农业及农产品深加工基地；在二产方面，打造有色金属及金属加工、运输设备及零部件、基础化工及精细化工、轻纺造纸及食品医药、能源电力及节能设备基地；在三产方面，打造商贸物流及现代物流、文化旅游及休闲度假基地。实现经济小市向经济大市的转变，实现经济发展由以量的增长为主向主要依靠科技进步、劳动者素质提高和管理创新的转变。

　　——今后五年，我们要大力发展文化事业文化产业，实现先进文化大发展。充分发挥文化引导社会、教育人民、推动发展的作用，把聊城潜在的文化影响力转变为现实的城市竞争力，把丰富的历史文化资源转变为现实生产力。社会主义核心价值体系深入推进，覆盖城乡的公共文化服务体系不断完善，文化事业全面繁荣，文化产业快速发展，社会文明程度明显提升，争创全国文明城市。“江北水城·运河古都”城市品牌进一步打响，城市文化影响力显著增强，成为内聚合力、外具魅力、特色鲜明、近悦远来的文化名城。

　　——今后五年，我们要着力加快新型城镇化进程，实现城乡面貌大变化。坚持统筹城乡发展，积极构建布局合理、规模适度、功能互补、城乡一体的城镇体系，城镇化率力争达到50%左右，城乡面貌、功能品质实现新的改善。着眼于提升聊城在省会城市群经济圈中的影响力和竞争力，加快中心城区建设，打造人口过100万、面积过100平方公里的“双百”大城市；积极构建以市城区为中心、茌平和东阿为支点的大三角地带，主动承接济南的辐射；抓好济聊一级公路、城际铁路等重大交通设施建设，实现聊城融入济南1小时经济圈。加强县（市）城区建设，完善城市功能，提高管理水平。有序建设小城镇和新农村，实现基本公共服务全覆盖。

　　——今后五年，我们要着力加强生态文明建设，实现生态环境大改善。以建设生态文明市为目标，加强绿色聊城建设，抓好大气和水污染治理，推进资源节约，发展循环经济，基本形成绿色低碳发展模式。在“十一五”的基础上，万元生产总值能耗降低17%，化学需氧量、氨氮排放总量分别减少12.7%和14.8%，二氧化硫、氮氧化合物排放总量分别减少17%和18%，森林覆盖率达到33%，生态质量明显提高，让聊城的天更蓝、水更清、空气更清新、环境更优美。

　　——今后五年，我们要着力保障和改善民生，实现人民生活大提高。坚持富民优先，更加关注民生建设，五年城镇居民人均可支配收入、农民人均纯收入年均增长13%，社会消费品零售总额年均增长17%。城乡就业保持较高水平，社会保障体系和公共服务体系进一步完善，社会事业适度超前发展。社会管理新格局基本形成，民主法制建设不断加强，公民权益得到依法保障，社会更加安全、和谐、稳定。

　　各位代表，经过五年的不懈奋斗，到2016年，全市生产总值将突破3500亿元，地方财政收入突破200亿元，城镇居民人均可支配收入达到3.8万元，农民人均纯收入达到1.4万元，聊城将成为现代产业体系基本形成、综合实力显著增强的城市，成为城乡区域协调发展、生活环境优美宜居的城市，成为社会更加和谐稳定、人民群众幸福感普遍增加的城市，成为社会文明程度显著提高、文化影响力显著增强的城市，一个经济繁荣、人民富裕、环境优美、社会和谐的新聊城一定会在鲁西大地蓬勃崛起！

　　三、2012年的目标任务和工作重点

　　今年是实施“十二五”规划承上启下的重要一年，我们党将召开十八大，做好今年的政府工作具有特殊重要的意义。我们要按照今后五年的目标任务，全面贯彻中央“稳中求进”的工作总基调，深入开展“争先进位年”活动，以加大优质高效投入、加强自主创新和节能减排、深化改革开放、保障改善民生为着力点，全面做好稳增长、控物价、调结构、惠民生、抓改革、促和谐的各项工作，努力保持经济又好又快发展，保持物价总水平基本稳定，保持社会和谐稳定，以优异成绩迎接党的十八大胜利召开。主要预期目标是：生产总值增长12%，地方财政收入增长18%，固定资产投资增长22%以上，社会消费品零售总额增长17%，实际利用外资增长20%以上，外贸进出口增长15%以上，城镇登记失业率控制在4%以内，城镇居民人均可支配收入、农民人均纯收入均增长12 %以上，居民消费价格涨幅控制在4%左右，人口自然增长率控制在7.3‰以内，节能减排指标全面完成省下达的任务。

　　工作中，重点抓好以下七个方面：

　　（一）突出抓好重点项目建设，努力加大优质高效投入。在扩大消费需求、保持物价总水平基本稳定的同时，把加大优质高效投入作为扩总量、转方式、调结构的突破口和着力点，围绕打造“一五二”产业基地，进一步加大投入力度，确保全市固定资产投资达到1300亿元。一是实施重点项目“双百工程”。重点抓好100个工业项目、100个服务业和其他项目建设，全年完成投资800亿元。着力优化投资结构，积极谋划科技含量高、地方财政贡献大、耗能占地少、环境污染轻、吸纳就业多的项目，做好济聊城际铁路、聊泰铁路、郑济客运专线、军民合用机场、青兰高速、煤炭资源开发等项目的前期工作。建立重点项目推进工作联席会议制度，加强组织协调，确保在项目数量、项目质量和建设进度上实现新的突破。二是加强各类园区建设。把园区作为项目建设、工业发展的主战场，坚持高起点规划，加强基础设施建设，全面落实“镇园合一”管理体制，力争所有开发区在全省实现位次前移， 2个开发区进入全省综合排名20强。三是努力解决发展“瓶颈”。积极推进银企对接，稳步开展股权交易，加大企业上市工作力度，推动企业债券发行，为企业发展、项目建设提供资金支持。加大向上争取、工矿废弃地复垦、土地增减挂、土地综合整治、地下空间开发利用工作力度，坚持土地指标差别化管理，提高土地集约利用水平。各位代表，围绕重点项目建设，我们要进一步调动全市上下的积极性，全面营造抓重点项目建设、支持重点项目建设的浓厚氛围，确保实现大的突破。我们相信，只要我们坚持一年一年地抓下去，由量变到质变，聊城就一定能够争先进位、跨越发展！

　　（二）全面落实农业产业调整振兴计划，推进农业发展农民增收。把实施农业产业调整振兴计划作为农业农村工作的抓手，以打造生态农业及农产品深加工基地为目标，突出抓好100个农业重点项目建设，加快构建现代农业体系。一是实施吨粮市建设工程。加快全国新增千亿斤粮食产能规划聊城项目区建设，加大农业综合开发力度，深入开展高产创建活动，在稳定播种面积的基础上，依靠科技进步提高单产和品质，确保粮食产量稳定在100亿斤以上，用2至3年的时间建成吨粮市。二是大力推进农业结构调整。鼓励群众种植附加值高、经济效益好的经济作物和园艺产品，发展畜牧和水产养殖，推动蔬菜和食用菌、畜牧和水产、林果生产及加工三大支柱产业规模进一步扩大。推进区域化布局、标准化生产、规模化种植，抓好农业标准化整体推进示范县、示范区建设，打造绿色有机和无公害农产品品牌。三是提高农业产业化水平。以100个农业产业化示范基地为重点，加快建设畜禽及饲料加工、蔬菜加工保鲜、林果及木材加工、水产加工、粮油加工等五大加工体系，培植壮大农业龙头企业，新增市级规模以上龙头企业30家。大力发展农民合作组织， 扶持壮大100家重点合作社。加快聊城农产品大市场等重点市场建设，积极推进农超对接、农企对接、农餐对接，进一步完善农产品流通体系。四是强化农业基础设施建设。抓好南水北调聊城段、平原水库和小农水重点县项目建设，实施徒骇河治理，加快位山、彭楼、陶城铺、郭口等灌区续建配套与节水改造工程建设。大力建设“旱能浇、涝能排”高标准农田，新增改善灌溉面积70万亩。鼓励农民购置大中型农机具，推进农机服务市场化和产业化，提高农业机械化水平。五是加快推进农业科技进步。实施农业产业科技创新工程，以良种培育、节水灌溉、农机装备、新型肥药为重点，推进产学研、农科教结合，提高自主创新能力。健全农技推广、动植物疫病防控、农产品质量安全监管等公共服务机构，强化基层公益性农技推广服务。继续深入实施科技特派员工程，加强科研成果推广。加大教育培训力度，大力培训农村实用人才。

　　（三）加快实施“4455”工程，推进工业做大做优。把实施“4455”工程作为工业经济发展的抓手，以打造“五大基地”为目标，努力加快新型工业化进程。一是大力推进“千亿产业园区”建设。把四大“千亿产业园区”作为培植四大战略性支柱产业的着力点，理顺管理体制，完善发展规划，加大招商引资和项目建设力度，加快发展步伐。建立健全镇企园合一的管理体制，积极推进工农城乡一体化，努力形成工业化、城镇化和农业现代化同步推进、和谐发展的新格局。二是抓好战略性新兴产业培育发展。设立战略性新兴产业发展基金，加大对新能源、生物医药、节能环保和高端装备制造等产业的支持力度，鼓励新企业建设和现有企业膨胀规模，新口径高新技术产业产值占规模以上工业比重提高1个百分点以上。三是推动民营经济发展实现突破。把发展民营经济作为富民强市的重大举措，设立中小企业发展专项资金，充分调动乡村两级的积极性，确保全市平均每个乡镇民营经济税收增加500万元、固定资产投资过亿元。以五大特色产业为重点，大力培植产业集群，加强服务体系建设，打造区域品牌。抓好创业辅导基地和公共服务平台建设，推进全民创业，帮助小微企业减负增效，努力为民营经济发展营造良好环境。四是促进骨干龙头企业发展壮大。加大扶持力度，鼓励50户骨干龙头企业加强项目建设、加大研发投入，进一步膨胀规模、提升竞争力，推动有条件的企业向千亿元大企业集团迈进。五是强化科技创新和节能减排。设立新产品研发和科技创新基金，鼓励企业增加研发投入，增强核心竞争能力。抓好九州国际高科园、西安交大聊城科技园、聊城有色金属研究院等科技创新平台建设。加快传统产业转型升级，抓好54个重点技改项目建设，鼓励企业引进先进技术和装备。深入推进“两化”融合，加快浪潮集团聊城信息产业园、云计算中心建设。深化同高校院所的合作，积极搭建与聊城大学的合作平台。深入实施人才强市战略，落实“533”高端人才引进计划，发展壮大高技能人才队伍。实施质量兴市战略，推进鲁西质检中心建设，争创省长质量奖和国家、省名牌产品。坚持依靠节能减排拓展发展空间，加快淘汰落后产能，做好重点县（市、区）、重点行业、重点企业节能工作；以大气和水污染治理为重点，建设燃煤电厂脱硝工程和重点企业排污整治工程；做好代表山东省迎接国家海河流域核查工作，确保再次取得优异成绩。

　　 （四）大力发展商贸物流、文化旅游，推动服务业跨越发展。把加快服务业发展作为转方式、调结构的重要内容，以打造商贸物流及现代物流、文化旅游及休闲度假两大基地为目标，力争完成服务业投资400亿元，增加值占生产总值比重提高2个百分点左右。一是大力发展商贸物流业。围绕打造冀鲁豫交界地区商贸流通中心城市，加快北部物流园区建设，推进盖氏物流、豪德物流等项目建设，加快构建商贸物流中心城市框架。大力培育商贸物流企业集团和现代批发市场，做好重点企业主辅业务分离工作，鼓励发展第三方物流。积极推进“万村千乡”市场工程，完善农村现代流通体系。二是加快发展文化旅游业。围绕打造江北文化旅游和休闲度假目的地城市，加快推进“江北水城·运河古都”旅游度假区、马颊河（世界运河文化之窗）生态旅游度假区、古漯河生态旅游度假区、阿胶养生文化苑、水浒文化旅游区等60个重点项目建设，打造大型文化旅游综合体。启动旅游集散中心建设，推进旅游产品整合，搞好对外宣传营销，大力拓展旅游市场。加快发展文化产业，积极推进市京剧院、豫剧院和杂技团改革，深化聊城报业传媒集团和广电集团改革，引进、培育新兴文化产业，打造特色文化产业品牌，推动文化旅游互促互动、繁荣发展。三是积极发展其他服务业。支持发展金融业，优化金融生态环境，积极争取辖外股份制商业银行来我市设立分支机构，推进农村信用社银行化改革，规范民间融资中介机构发展，做好小额贷款公司、村镇银行的设立工作，规范发展各类保险机构。调整优化住房供应结构，扩大中小套型、中低价位住宅供应。鼓励发展农村服务、信息服务、社区服务、中介服务等服务业。各位代表，今年我们专门设立了服务业发展专项资金，要切实加大支持力度，努力在服务业发展上实现大的突破！

　　（五）统筹推进城乡建设，加快新型城镇化进程。把加快新型城镇化进程作为支撑新型工业化、改善群众生活条件的重要途径，全面加强城乡建设，确保城镇化率提高2个百分点以上。

　　大力推进百万人口大城市建设。按照“一城五区”总体框架，以“两城一河”为重点，以城建重点项目为抓手，全面加强城市规划建设和管理，推进中心城区“东扩、南展、北延”，加快构建大城市格局。重点抓好八大工程：一是古城保护与改造。完成四关大街建设，启动七贤堂、县衙等8处景点和西南、东南片区建设。二是徒骇河世界运河（建筑）博览园。完成滨河大道建设，实施湖南路至新南环路河道治理和沿岸景观绿化。三是南部新区重点项目。完成体育公园主体工程，启动恒大集团南部城市综合体建设，开展湖南湖清淤和东南湖沿岸整治。四是北部商贸物流园区。完成物流园区一期、农产品大市场一期，启动豪德物流建设。五是东部工业新城。完成中央商务区会展中心、五星级酒店主体、九州生态公园铺装和部分景观项目。六是市政基础设施。实施昌润路南延等17条道路建设，加快中水回用工程、生活垃圾焚烧发电厂建设。七是园林绿化。实施城市绿荫行动，加强道路绿化改造，抓好城市出入口、城乡结合部和主要道路接点景观建设，全面提升广场、公园、单位庭园绿化水平。八是城市综合整治。抓好11条主次干道、40条背街小巷的整治提升。坚持疏堵结合、以疏为主，抓好步行街、早夜市建设。加强市政基础设施维护、园林绿化养护和环境卫生管理，全面提升城市形象。各位代表，我们要通过“八大工程”的实施，进一步拉大城市框架、完善城市功能、凸显水城特色，“一年一大步，三年大变样”，为建设百万人口大城市奠定坚实基础！

　　全面抓好县（市）城区、小城镇和新农村建设。全面调动县、乡、村三级加快发展的积极性，以经济的快速发展带动城乡建设，加快新型城镇化进程。加强县（市）城区建设，强化规划龙头作用，抓好基础设施建设，完善提升城市综合功能；把大企业带动、产业集群培育、园区发展与城镇建设紧密结合起来，着力加快县域经济发展，增强对经济发展的承载力、对农村人口的吸纳力。加大中心镇建设力度，落实扩权强镇各项措施，重点扶持30个中心镇发展，完善基础设施和公共服务，培植发展特色产业，加快规模膨胀。按照城乡一体化发展的要求，加强农村社区建设，引导更多的农村适度集中建设住房，推动农村新居建设常态化。依托现有优质水源，以城乡供水同网、同质为目标，推进城乡供水一体化，逐步让农村群众用上优质水、健康水。加强农村道路、垃圾处理、污水处理等基础设施建设，抓好农田林网、路网和水系生态绿化，进一步改善农村人居环境。

　　（六）进一步深化改革扩大开放，增强经济社会发展活力。把改革开放作为实现争先进位、跨越发展的强大动力，坚定不移地加以推进。一是积极推进各项改革。进一步深化国有企业改革，积极引进战略投资者和民间资本，推进国有企业股权多元化；加大整合重组力度，盘活国有资产，促进国有产权合理流动。加强国有资产监管，规范县（市、区）国有资产监管行为，完善外派监事会和外派财务总监制度。分类推进事业单位改革，在全面清理规范的基础上，基本完成事业单位分类工作。稳妥推进土地承包经营权流转市场建设，基本完成覆盖农村集体各类土地的所有权登记颁证工作。二是全面提升开放型经济发展水平。着力培育出口产品品牌，抓好莘县、阳谷、冠县三个出口农产品示范区建设。深化同日韩、港澳台的合作，突出抓好骨干龙头企业、战略性支柱产业、各类产业园区利用外资工作，推动企业在新加坡、香港等地上市，努力在利用外资的数量和质量上实现突破。加强“海外聊城”建设，鼓励企业开发境外资源、构建境外营销网络、建设境外工业园区、开展对外劳务合作。全面加强区域经济合作，积极融入省会城市群经济圈、加入中原经济区，主动对接山东半岛蓝色经济区、黄河三角洲高效生态经济区，加强与环渤海经济区的合作交流。各位代表，今年我们要继续开展招商引资“百日会战”，进一步加强领导、广泛发动，主攻项目落地，努力在招商引资总额、项目规模、项目层次、招才引智等方面实现新的突破，确保到位资金达到500亿元以上。

　　（七）着力保障改善民生，保持社会和谐稳定。把保障改善民生、创造聊城人民的幸福生活作为政府工作的出发点和落脚点，进一步加大投入，让人民群众得到更多实惠。

　　切实做好民生工作。围绕解决关系城乡百姓切身利益的困难和问题，重点办好10件民生实事：一是扩大就业。扶持就业容量大的现代服务业、创新型科技企业和小微企业发展，抓好高校毕业生就业见习基地、创业孵化基地建设，实施千个公益性岗位开发计划，落实职业培训补贴制度，开展创业助推行动，确保新增城镇就业6万人，其中失业人员再就业2万人、帮扶困难群体就业4000人，实现城乡“双零家庭”动态消零，在外务工人员保持在110万人以上。企业最低工资标准提高到950元，增长18.8%。二是社会保障体系完善。做好城乡居民养老制度衔接，把“五七工”、“家属工”和未参保集体企业退休人员纳入基本养老保险范围，提高医疗、失业、工伤、生育保险市级统筹水平。继续增加企业退休人员养老金，平均增长10%以上；农村低保标准提高到每人每年1800元，城市低保标准进一步提高；政府对新农合和城镇居民医疗保险补助标准提高到每人每年240元。三是保障性安居工程建设。新建廉租住房200套、公共租赁住房3000套、经济适用住房1000套、限价房4550套，棚户区改造6663套。四是加强社会救助。新建标准化特殊教育学校1处，市儿童福利院和县级福利中心力争年内投入使用，实行医疗救助“一站式”结算县级全覆盖，提高大病医疗救助水平，对0—6岁残疾儿童实施公益性抢救康复。五是教育条件改善。围绕促进公平、提高质量，进一步加大教育投入，促进各级各类教育全面发展。每个乡镇建设一处中心幼儿园，对孤儿、家庭困难和残疾儿童入园每人每年资助1200元。加快推进中小学办学条件标准化建设工程、校舍安全工程和农村中小学“211”工程，新建重建农村中小学校舍17万平方米，将农村初中和小学家庭困难寄宿生生活补助标准提高25元；新增城区小学教室120个，班额控制到50人以下。完成聊城高级财经职业学校和聊城高级工程职业学校新校区建设。深化同聊城大学、山东工程技师学院等驻聊高校的合作，支持聊城职业技术学院、东昌学院加快发展。促进民办教育健康发展。六是文体设施建设。抓好市民活动中心、市图书馆新馆、市豫剧院和县（市、区）文化场馆建设，推进国有博物馆、纪念馆、图书馆、文化站等向社会免费开放。完成体育公园主体工程，秋季举办第二届全市运动会。七是医疗卫生改善。扩大公立医院改革，逐步将村卫生室、城市社区卫生服务站纳入基本药物实施范围。开展公益性防病健身宣传，提高群众健康意识。继续实施农民健康服务行动，对农村贫困家庭的先天性心脏病儿童分步实施免费诊治。全力做好传染病防控等突发公共卫生事件应急处置工作，确保不出现暴发流行。八是实施“百村扶贫攻坚”计划。加大财政投入，全面推进产业扶贫、科技扶贫、政策扶贫，确保贫困村农民人均纯收入增幅高于全市平均水平。九是城乡居民生活条件改善。完成望湖安置小区二期工程，实施19个城中村改造，新增集中供热面积100万平方米、管道燃气用户1万户；完成既有建筑节能改造30万平方米、太阳能一体化建筑40万平方米。改进城建拆迁方式，集中建设经商场所，努力做到农民失地不失业、生活有保障。实施村级公路网化工程、县乡老油路升级改造，新建改造农村公路300公里，抓好农村公路维护，开展干线公路和农村公路路域环境综合整治。解决15万人饮水安全问题。十是食品安全。健全综合协调机构，理顺监管职能，加强动态监管和源头治理，深入开展专项整治，全面提高食品安全保障水平。

　　着力塑造聊城新形象。各位代表，市第十二次党代会提出，要着力塑造“自豪、创业、包容、奋进”的聊城形象。这是市委充分发掘聊城文化的优良传统，结合时代发展的内在要求，向全市广大党员干部和600万人民提出的新要求。各级政府要深入开展“塑造聊城形象从我做起”主题活动，引导广大干部群众进一步解放思想、提高素质、树立形象，做自豪的聊城人、创业的聊城人、包容的聊城人、奋进的聊城人！

　　全面推进和谐社会建设。积极发展文化事业，实施艺术创作精品工程，深入挖掘传统文化，抓好运河申遗工作，采取多种形式丰富群众文化生活。坚持和稳定计划生育政策，健全完善利益导向机制，加强流动人口管理服务，加大出生人口性别比治理。健全优抚保障服务体系，强化退役士兵就业技能培训和服务，进一步提高双拥优抚安置水平。理顺食品药品监管职能，完善责任体系，严格行政执法和责任追究。加强安全生产基层基础工作，抓好对重点行业、重点领域的专项整治，坚决遏制重特大安全事故发生。深化“平安聊城”建设，扎实开展全民普法教育，加强社会治安综合治理，依法打击各类违法行为，保持社会稳定。继续做好减灾救灾、社会科学、民族宗教、外事侨务、残疾人、慈善、对台、油区、人防、档案、史志、气象、测绘、防震等项工作。

　　四、进一步加强政府自身建设

　　各位代表，做好政府工作，加强自身建设是基础和前提。我们要始终按照为民、务实、清廉的要求，努力建设人民满意的政府。

　　（一）努力开创勇于创新、争先进位的新局面。我们要进一步解放思想、自我加压、敢于争先。各级政府、各部门和单位都要分析研究在全省的位次，明确争先进位的目标，并作为政府目标责任管理的重要内容。切实增强创新意识，认真做好结合的文章，不断创新工作思路、工作方式、工作方法。努力营造宽松环境，激励创新、支持改革，容许尝试、宽容失败，形成敢闯敢试、奋勇争先的浓厚氛围。

　　（二）全面形成转变作风、狠抓落实的新风貌。切实加强调查研究，领导同志带头搞调研，政府组成人员每年拿出不少于2个月的时间深入基层。坚持讲实话、办实事、求实效，依法推进统计方法制度改革，加强统计体系建设，坚决反对形式主义、弄虚作假。严格执行转变作风、狠抓落实“八项制度”，每项工作都要明确工作目标、责任主体、责任人、完成时限，并严格考核奖惩；对重要工作、重大项目，领导同志要现场办公、帮助解决问题，责任单位要公开承诺、接受社会监督，督查机构要跟踪督办、全程督查，确保扎实推进、取得实效。加强对重大决策执行情况、重点工程建设情况、重要事项落实情况的效能监察，坚决治“庸”、治“懒”、治“散”、治“软”，提高工作效率，促进工作落实。

　　（三）加快构建依法行政、规范有序的新机制。以贯彻落实《山东省行政程序规定》为抓手，规范行政权力运行，加快法治政府建设。严格执行人大及其常委会的决议、决定，主动接受人民政协的民主监督，认真做好人大代表建议和政协提案办理工作。加快行政审批制度改革，进一步清理、减少行政许可及审批事项，推行网上审批，强化联审联办，扩大全程代办范围，推进市、县两级行政服务中心统一运行。建立健全重大行政决策程序，完善公众参与、专家论证与政府决定相结合的决策机制。深化行政执法体制改革，推进行政处罚权相对集中，加强对行政裁量权的管理，细化执法标准，严密执法程序。积极推进行政复议权相对集中，依法运用行政调解等方式化解社会矛盾。

　　（四）进一步树立执政为民、廉洁勤政的新形象。牢固树立群众观念，经常深入基层、深入群众，尽可能多地为群众办实事、办好事，为群众排忧解难。全面推进政务公开，所有面向社会服务的政府部门都要实行办事公开制度。抓好政府门户网站、便民服务网络平台建设，更好地方便群众办事。认真办好市长公开电话，扎实开展政风、行风评议和社情民意调查，畅通群众诉求渠道。抓好对群众反映强烈问题的治理，坚决纠正损害群众利益的不正之风。坚持“一岗双责”，认真落实廉政建设责任制，加强惩治和预防腐败体系建设。推行廉政风险防范管理和科技防腐，完善行政权力、公共服务公开透明运行机制，加强监察、审计等专门监督。加大对违纪违法案件查处力度，严厉查处腐败行为。加强对各级公务员的教育，不断增强为民服务意识，提高拒腐防变能力，努力打造一支廉洁自律、敬业奉献的公务员队伍。

　　各位代表，百舸争流、千帆竞进，我们一定要以更加开阔的视野、更加昂扬的斗志、更加扎实的作风，踏踏实实地干好工作，向全市人民交一份满意的答卷。让我们更加紧密地团结在以胡锦涛同志为总书记的党中央周围，在市委的坚强领导下，解放思想、开拓创新、奋力拼搏，为全面建设生态型强市名城、创造聊城人民更加美好的幸福生活而努力奋斗！
